# *In vitro* and *in vivo* effects of insulin-producing cells generated by xeno-antigen free 3D culture with RCP piece

**DOI:** 10.1038/s41598-019-47257-7

**Published:** 2019-07-24

**Authors:** Tetsuya Ikemoto, Rui Feng, Shu-ichi Iwahashi, Shinichiro Yamada, Yu Saito, Yuji Morine, Satoru Imura, Munehide Matsuhisa, Mitsuo Shimada

**Affiliations:** 1 Department of Digestive and Transplant Surgery, Tokushima University, 3-18-15 Kuramoto, Tokushima, 770-8503 Japan; 2 Diabetes Therapeutics and Research Center, Tokushima University, 3-18-15 Kuramoto, Tokushima, 770-8503 Japan

**Keywords:** Type 1 diabetes, Stem-cell research

## Abstract

To establish widespread cell therapy for type 1 diabetes mellitus, we aimed to develop an effective protocol for generating insulin-producing cells (IPCs) from adipose-derived stem cells (ADSCs). We established a 3D culture using a human recombinant peptide (RCP) petaloid μ-piece with xeno-antigen free reagents. Briefly, we employed our two-step protocol to differentiate ADSCs in 96-well dishes and cultured cells in xeno-antigen free reagents with 0.1 mg/mL RCP μ-piece for 7 days (step 1), followed by addition of histone deacetylase inhibitor for 14 days (step 2). Generated IPCs were strongly stained with dithizone, anti-insulin antibody at day 21, and microstructures resembling insulin secretory granules were detected by electron microscopy. Glucose stimulation index (maximum value, 4.9) and MAFA mRNA expression were significantly higher in 3D cultured cells compared with conventionally cultured cells (*P* < 0.01 and *P* < 0.05, respectively). The hyperglycaemic state of streptozotocin-induced diabetic nude mice converted to normoglycaemic state around 14 days after transplantation of 96 IPCs under kidney capsule or intra-mesentery. Histological evaluation revealed that insulin and C-peptide positive structures existed at day 120. Our established xeno-antigen free and RCP petaloid μ-piece 3D culture method for generating IPCs may be suitable for clinical application, due to the proven effectiveness *in vitro* and *in vivo*.

## Introduction

Islet transplantation (ICTx) is one of the therapeutic options for patients with type 1 diabetes mellitus and can remove the need for insulin injections and associated complications^1,2^. However, ICTx still faces several obstacles, such as the requirement of a large islet yield^2^, the fragility of the isolated islets^3^ and severe donor shortages in some counties, such as Japan^4^, which need to be addressed before widespread clinical use.

To solve these issues, we have focused on stem cells for generating insulin-producing cells (IPCs). We investigated adipose-derived stem cells (ADSCs) as a new cell source^5,6^, not only for their trophic effects but also for their multi-potency. We focused on these cells because ADSCs can be obtained from the patient’s own fat tissue under local anaesthesia, and auto-ADSC transplantation has fewer ethical problems compared with the use of induced pluripotent stem or embryonic stem cells. Furthermore, transplanted induced pluripotent stem cells or transfected embryonic stem cells can harbour DNA damage, and thus carry a high risk of cell transformation and carcinogenesis^7^. ADSCs do not show these limitations and can be readily applied in clinical trials^8,9^. Moreover, some reports indicate that ADSCs show superior potential for differentiation into various cell types^10^, that sufficient cell numbers can be easily obtain with ADSC compared with other types of stem cells, for example, bone marrow derived stem cells^11^.

We previously established an accelerated IPC generation protocol from ADSCs using histone deacetylase inhibitor (HDACi), using modified protocols from previous studies^12–15^. HDAC inhibition is reported to be a strong driver of pancreatic cell lineage progenitors^16^. Therefore, we investigated the use of an HDACi (valproic acid) for acceleration of IPC formation and established a rapid and easy two-step protocol^17^. Although we achieved simplification and shorten culture duration, some hurdles still remained for clinical application, such as the presence of xeno-antigens, poor cell expansion rate and unexamined *in vivo* effectiveness.

The three-dimensional (3D) culture system has demonstrated many advantages for cell expansion and cell transplantation. Various 3D culture systems have been established, such as those that use matrigel, scaffolds, special dishes and floating culture systems^18–21^. To improve our IPC generation protocol, we used the human recombinant peptide (RCP) petaloid μ-piece obtained from FUJI FILM Co. Ltd. This material showed advantages for islet cell culture and cell transplantation^22^ in a murine experimental model. Briefly, this RCP μ-piece supports the formation of a large cell cluster, named as a ‘CellSaic’ because cells and the RCP μ-piece are merged like a mosaic, without central necrosis. The CellSaic maintains cell viability *in vivo* when transplanted with target cells. Additionally, CellSaic is xeno-antigen free and clinical grade has been achieved^23^.

Here we report our established 3D culture system that is based on our easy and rapid two-step IPC generation protocol and free of xeno-antigen. We also demonstrate *in vitro* and *in vivo* results that support the potential clinical application of the generated IPCs.

## Results

### The RCP piece 3D culture system generates cells with IPC morphology

**Figure 1:**
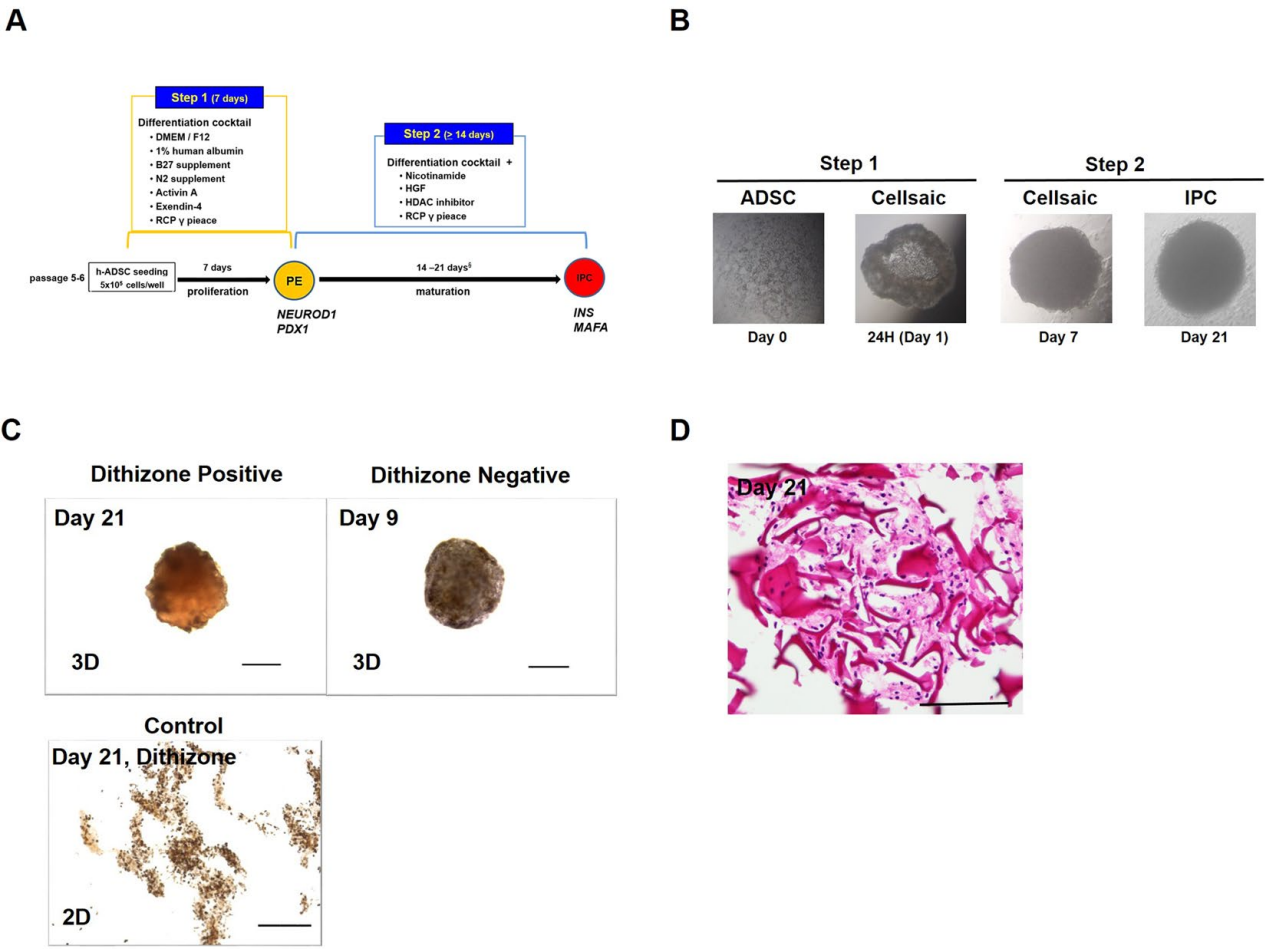
Our established protocol for generating insulin-producing cells in 3D culture using our two-step protocol. **(A)** Schematic showing the two-step protocol for generating IPCs from ADSCs. §: in the case of ordinary plate culture (conventional 2D monolayer culture). PE: pancreatic endoderm, IPC: insulin-producing cell. **(B)** Images of cell formation at each step are shown. ADSCs rapidly formed a cluster in 24 h and the cluster was maintained until day 21 (step 2). **(C)** The generated cell cluster was strongly stained with dithizone dye at day 21 after the start of step 1 (upper left), but was not well stained at day 9 (upper left) and 2D cultured control at day 21 (bottom). Scale bar, 100 μm. **(D)** Formation of a ‘CellSaic’ with viable cells and RCP petaloid μ-piece. Scale bar, 100 μm.

We generated IPCs from ADSCs under 3D (with μ-RCP piece) or conventional conditions using our two-step protocol (Fig. 1A). Morphological changes were examined every two days during the culture, and representative images at day 1, day 7, and day 21 are shown in Fig. 1B. A sphere-like cell cluster formed within 24 h after initiation of the 3D culture and maintained formation until day 21. These cells were strongly stained with dithizone at day 21 (Fig. 1C, upper right), but not well stained at day 9 (Fig. 1C, upper left) or in 2D culture (as control) at day 21 (Fig. 1C, lower), and cell formation and nuclei were maintained and showed good cell condition without necrosis under a light microscope (Fig. 1D).

### Immunohistochemical staining showed that viable IPCs secreted insulin

**Figure 2:**
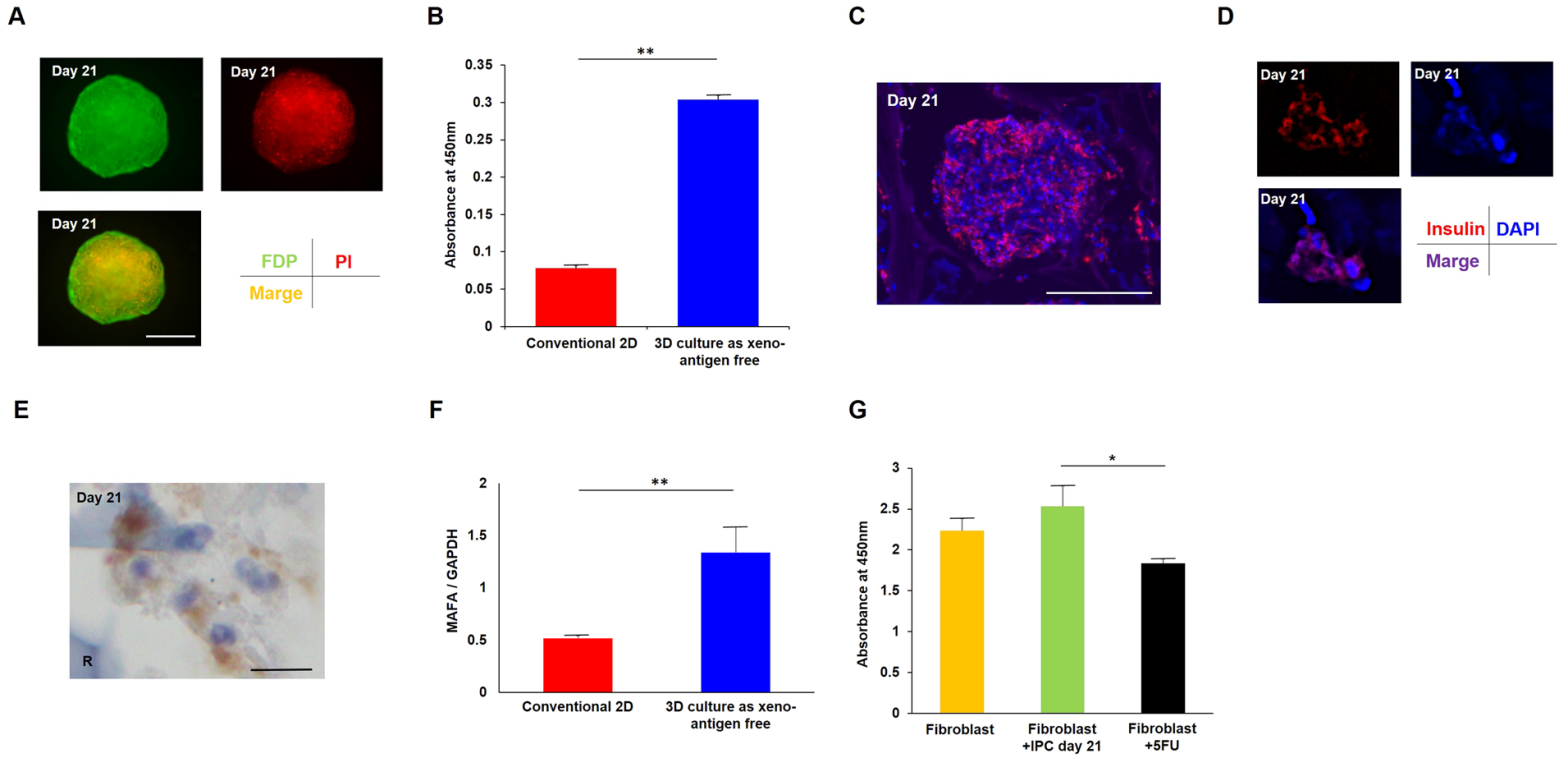
Cell quality of IPCs generated by the 3D cultured protocol was better than IPCs from conventional monolayer culture. **(A)** The cell viability of 3D cultured IPCs at day 21 was determined by FDP/PI staining. Green: FDP staining, red: PI staining, orange: merged image. Scale bar, 100 μm. **(B)** Absorbance at 450 nm showed significantly better cell viability of 3D cultured IPCs compared with IPCs from conventional 2D culture method. **P < 0.01, un-paired t-test. **(C)** Immunofluorescence of generated IPCs at day 21. Red: insulin, blue: DAPI. Scale bar, 100 μm. **(D)** The larger image shows the insulin-positive area was the cytoplasm. Red: insulin, blue: DAPI. **(E)** Light microscopy analysis also showed the cytoplasm of generated IPCs was positive for insulin staining. Scale bar, 10 μm. R: RCP petaloid μ-piece, stained blue. **(F)** MAFA mRNA expression was significantly higher in 3D cultured IPCs compared with IPCs from conventional 2D culture method by unpaired t-test. **P < 0.01. Error bar indicates standard deviation. **(G)** Generated IPCs did not show significant adverse effects on human dermal fibroblasts in cell toxicity, although 100 μM 5-FU addition showed significantly reduced viability. *P < 0.05, one-way ANOVA.

As determined by fluorescein diacetate/propidium iodide (FDP/PI) staining (Fig. 2A), the 3D cultured IPCs on day 21 showed significantly better cell viability compared with the 2D culture cells (*P* < 0.01, unpaired t-test, Fig. 2B). Moreover, the cytoplasm of 3D cultured IPCs showed positive staining for insulin in immunological fluorescence images (Fig. 2C,D). Light microscopy imaging also showed positive staining for insulin of the cytoplasm (Fig. 2E).

We next investigated mRNA expression of MAFA, a maturation marker of islet β-cells. The expression of MAFA mRNA in 3D cultured IPCs at day 21 was significantly higher than that of conventional 2D cultured IPCs at day 21 (*P* = 0.00449, unpaired t-test; Fig. 2F).

### Generated IPCs did not show significant toxicity

We also investigated the cell toxicity of generated IPCs by evaluating the effects on the proliferation rate of human fibroblasts. The co-culture of IPCs at day 21 did not show significant adverse effects on the proliferation rate of human fibroblasts compared with the control group, although 5-FU addition showed significantly reduced viability (P < 0.05, one-way ANOVA, Fig. 2G).

### Electron microscopy shows insulin secretion granule-like structures in 3D cultured IPCs

**Figure 3:**
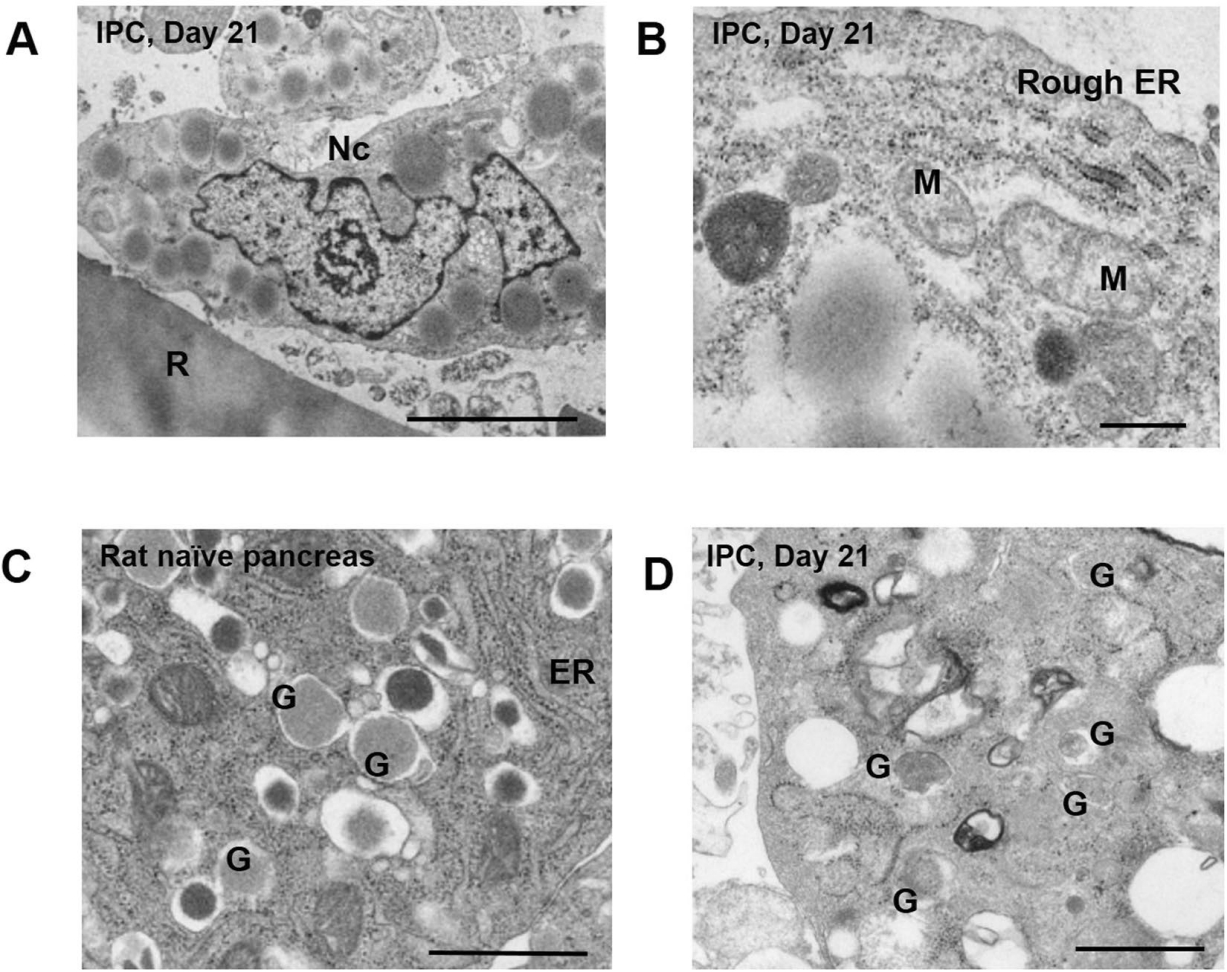
Electron microscopic images of microstructures in IPCs. **(A)** Generated 3D cultured IPCs at day 21 showed clear nuclei. Nc: nucleus, R: RCP petaloid μ-piece. Scale bar, 5 μm. **(B)** Generated 3D cultured IPCs at day 21 also showed organelles. M: mitochondria, ER: endoplasmic reticulum. Scale bar, 1 μm. **(C)** Control: section from naïve rat pancreas. Naïve rat islets show dense cystic microstructures. ER: endoplasmic reticulum, G: secretory granule. Scale bar, 1 μm. **(D)** The same microstructures morphologically resembling secretory granules existed in generated IPCs at day 21. G: secretory granule. Scale bar, 1 μm.

Electron microscopic analysis revealed that the nuclei of 3D cultured IPCs contained dense cystic microstructures (Fig. 3A) and organelles, such as rough endoplasmic reticulum and mitochondria (Fig. 3B). Moreover, these dense cystic microstructures in the generated IPCs morphologically resembled insulin secretion granules observed in normal islets from naïve rat pancreas (Fig. 3C,D).

### Generated IPCs show a good glucose stimulation index (SI)

**Figure 4:**
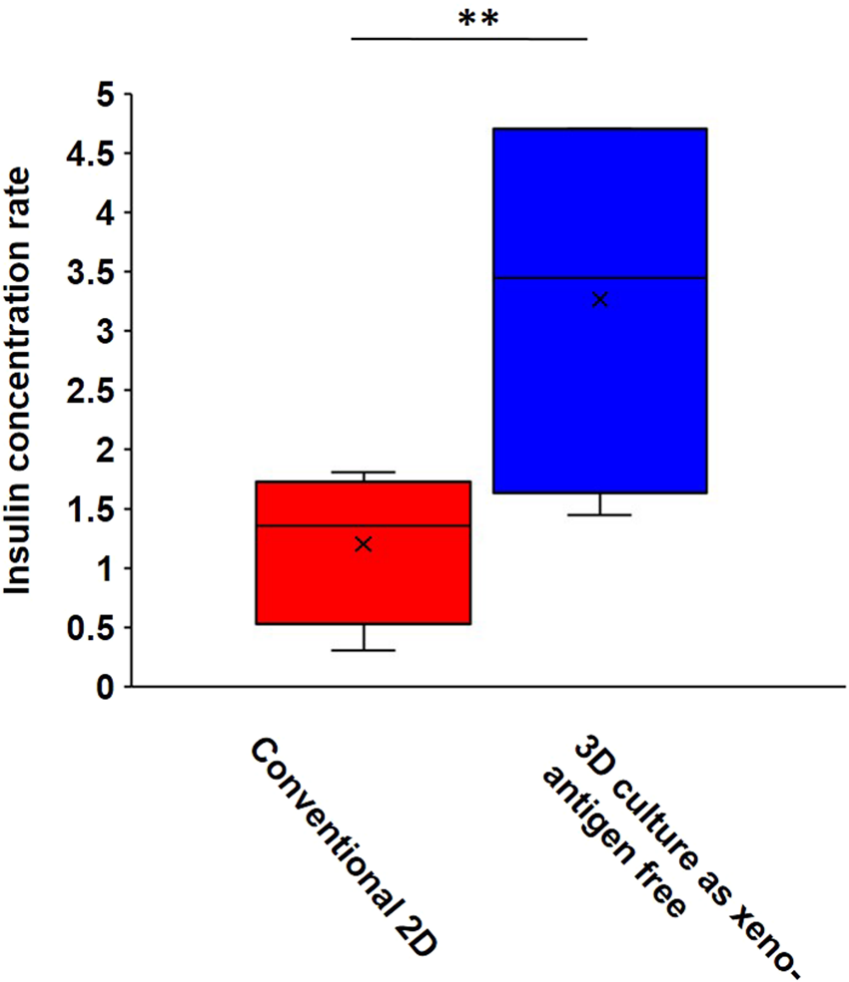
Glucose stimulation index of IPCs generated by the 3D cultured protocol was significantly higher than IPCs generated by conventional 2D culture. The lines in the boxes show the median values, and the error bar shows the standard deviation. Unpaired t-test, **P < 0.01.

The insulin concentration in the supernatant of IPCs at day 21 was increased when these cells were incubated in ‘low glucose’ medium (5 mM) for 1 h and then moved to ‘high glucose’ medium (45 mM) for 1 h. The average glucose SI of 3D cultured IPCs was significantly higher compared with conventional 2D cultured IPCs (*P* < 0.05, Welch’s test, Fig. 4). The maximum SI value in 3D cultured IPCs at day 21 was 4.9.

### Generated IPCs converted from a hyperglycaemic to a normoglycaemic state after transplantation

**Figure 5:**
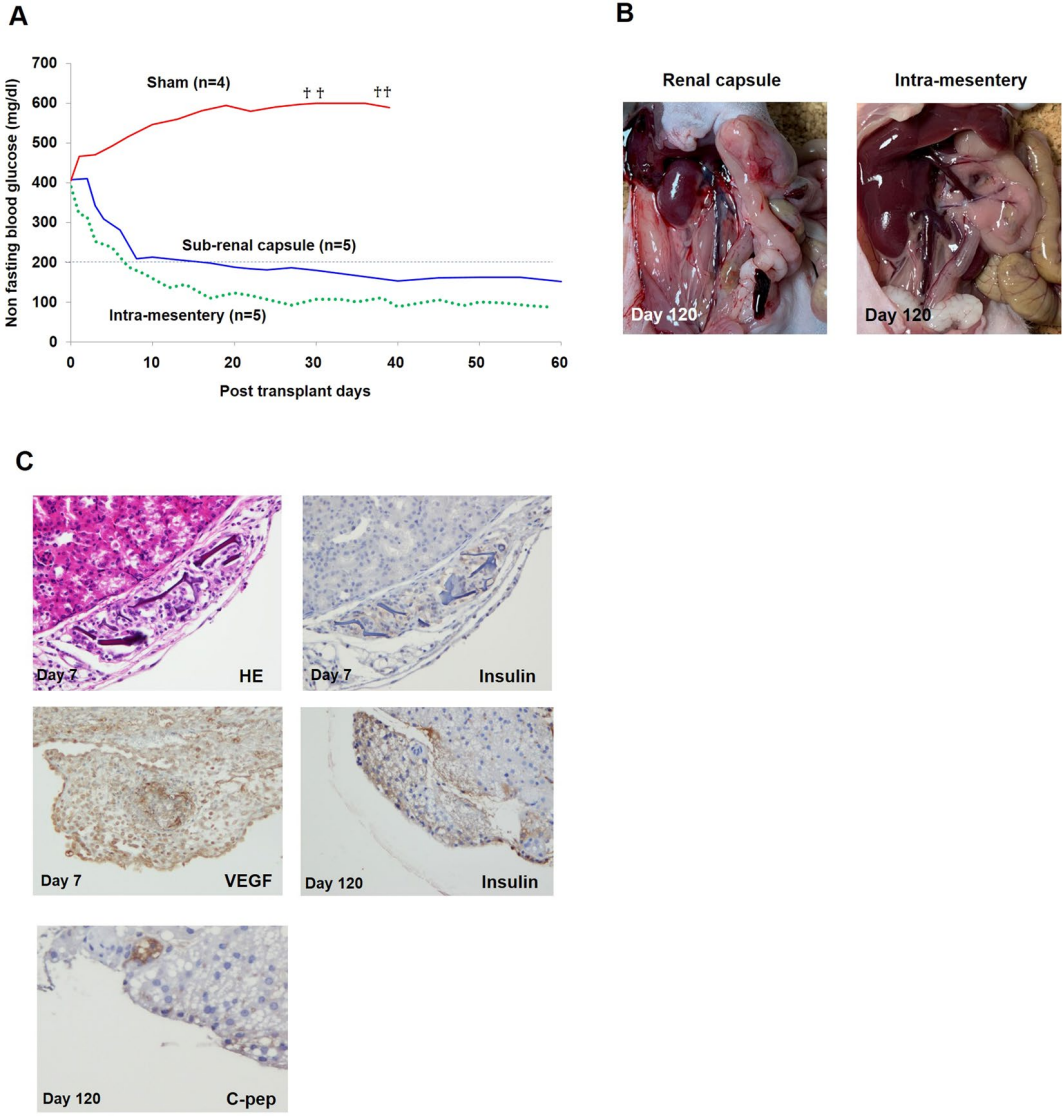
*In vivo* study. **(A)** Non-fasting blood glucose levels were decreased gradually after transplantation and measured below 200 mg/dl around 14 post-transplant days and maintained up to 60 post-transplant days in sub-renal capsule group (n = 5) and intra-mesentery group (n = 5). †: death. **(B)** No abnormal tumor formation was observed in each transplanted site at 120 post-transplant days. Left: around IPC transplanted right kidney, Right: mesentery. **(C**) Upper left. Transplanted CellSaic under kidney capsule at day 7. Central necrosis was not observed. Haematoxylin-eosin staining. Original magnification, ×400. Upper right. Continuous section of upper left was stained by anti-insulin antibody. Cytoplasm of IPC in CellSaic was strongly stained for insulin. Original magnification, ×400. Middle left. Transplanted IPCs at day 7 also showed strong expression of VEGF. Original magnification, ×400. Middle right. Transplanted CellSaic under kidney capsule at day 120. Central necrosis was hardly observed. Cytoplasm of IPC in CellSaic was strongly stained for insulin. RCP pieces were vanished, considered to have been absorbed. Original magnification, ×400. Lower. Some parts of this structure were strongly stained by C-peptide. Original magnification, ×800.

Non-fasting blood glucose levels of streptozotocin-induced diabetic nude mice decreased gradually in IPC transplantation groups, but not in the sham group. Levels were below 200 mg/dl around 14 post-transplant days and were maintained up to 120 post-transplant days (17 weeks) in the sub-renal capsule transplantation group (n = 5) and intra-mesentery transplantation group (n = 5). Hyperglycaemic state was not converted in the sham group (n = 4). The average non-fasting blood glucose levels of each group up to 60 post-transplant days are shown in Fig. 5A.

### Transplanted IPCs showed strong insulin and VEGF immunostaining

Macroscopic investigations for IPC-transplanted mice at day 120 post-transplantation revealed no macroscopic teratomas and carcinomas around transplanted sites (Fig. 5B). The kidney at day 7 and at day 120 were extirpated and investigated microscopically. Light microscopy imaging revealed that transplanted IPCs under renal capsule at day 7 (Fig. 5C, upper) and at day 120 show few central necrosis, and showed insulin staining by immunofluorescence (Fig. 5C, middle right). Some parts of this structure were strongly stained by C-peptide (Fig. 5C, lower left). Transplanted IPCs at day 7 also showed strong expression of VEGF (Fig. 5C, middle left).

## Discussion

Repeated ICTx is recommended to increase the possibility of achieving insulin-free status, and more effective immunosuppression for long-lasting effects of ICTx is expected even though ICTx has been undergone clinically in Europe and North America. However, transplant surgeons in severe donor-shortage counties such as Japan have struggled to find a new cell source for ICTx. To solve these and other urgent issues for ICTx^2,24,25^, regenerative medicine has been a subject of focus and thus the multipotency abilities of stem cells have been investigated. We focused on ADSCs among mesenchymal stem cells, as these cells can be used for procurement with less invasiveness methods and ADSCs were reported to have more multipotency compared with other cell sources^26,27^.

We previously established an easy and rapid differentiation protocol of IPCs from ADSCs. Despite the promising preliminary results, the method showed some issues to be addressed before clinical application. These hurdles included a relatively poor cell expansion rate^12,28^, the requirement for elimination of xeno-antigen^29^, and evaluation of whether beneficial effects are realized in recipients^30^. Here we developed a 3D culture system with a RCP petaloid μ-piece to successfully generate effective IPCs that marked 65.6% of cell expansion rate (according to DNA quantity, data not shown) and reasonable SI value. Moreover, this RCP petaloid μ-piece is xeno-antigen free and is provided as clinical grade^22,23^. Our established two-step generation protocol is thus a xeno-antigen-free protocol. We also demonstrated the generation of effective IPCs *in vitro* with microstructures and showed they are viable insulin releasing cells by electron microscopy. Notably, the average diameter of these generated IPCs is relatively large, and thus further investigations are required to examine why central necrosis rarely occur, as well as the underlying mechanisms and *in vivo* pathological effects. Previous studies have shown that *in vitro* generated cells may not show ideal effects *in vivo*^30,31^. Indeed, the effectiveness of generated IPCs differentiated from same cell number with conventional culture (2D) was limited and unstable (data not shown). Pathological analysis suggests this might be due to poor angiogenesis compared with 3D cultured transplantation results. Therefore, we performed *in vivo* transplantation experiments with the IPCs generated by our 3D and xeno-antigen free protocol. The non-fasting blood glucose level of STZ-induced diabetic immunodeficient nude mice showed the conversion from hyperglycaemia to normoglycaemia in mice with IPCs transplanted in sites such as under kidney capsule or intra-mesentery, and not in mice with injection in intra-femoral muscle. We are currently performing experiments with more mice in each group. We speculate that the poor angiogenesis or destruction of transplanted IPCs might occur from mechanical compression (data not shown). The normalization speed and blood glucose level were superior in intra-mesentery IPC transplantation than those with the renal capsule approach, which may be due to the difference in angiogenesis as described above. This glycaemic state was maintained at least until 120 post-transplant days. According to our previous experiments^3,4,32,33^ and reported studies^34,35^, one human IPC has the same insulin release activity *in vivo* of three international equivalent freshly isolated human islets, and graft survivals and endocrine activity were equal to superior than that of islet transplantation. Moreover, ICTx requires allogenic multi-donors-one-recipient transplantation^36^, and generation from the patient’s own small subcutaneous fat tissue under local anaesthesia can undergo repeated auto-transplantation with few ethical problems^11^. Although we showed preliminary data for the risk of carcinogenesis and cell toxicity of generated IPCs, a preclinical trial will be required to compare the 3D generated IPCs with islet transplantation once several questions are addressed regarding the generated IPCs, such as toxicity tests, elimination methods for undifferentiated cells, the decision of prompt transplantation site and dividing cell batches^11,37,38^. To address requirements of governmental regulations, we are now planning higher accuracy cytotoxicity LDH assay, soft agar colony forming test as cell transformation assay *in vitro*, and *in vivo* assay using NOD/Shi-scid, IL-2Rγnull or NOD.Cg-*Prkdc*^*scid*^*Il2rg*^*tm1Wjl*^/SzJ mice for proof of concept acquisition. However, the elimination of immature cells is very important. Dithizone staining is used for the evaluation of cell maturation; however, this method is a relative and destructive^4,39^ evaluation method. Thus, we have investigated a non-destructive evaluation approach to distinguish immature IPCs using zinc ion concentration in the supernatant of cultured IPCs (manuscript submitted). Moreover, whether generated IPCs from the fat tissue of a type 1 diabetic patient undergo destructions when these IPCs are transplanted in autoimmune disease patients will require investigation^40^. Notably, our newly developed protocol to generate IPCs from ADSCs shows several advantages, such as xeno-antigen free, no genetic manipulation, effective culture system, low ethical problem from small fat tissue procurement under local anaesthesia to auto-transplantation, no immunosuppressive therapies and proven *in vivo* effects. Therefore, our strategy may be effective and useful in type 1 diabetes patients in the near future.

## Materials and Methods

### IPC generation protocol

IPCs were generated by 3D culture with our two-step protocol^17^. Briefly, this protocol is based on DeAmour *et al*.^12^ and we modified the previous protocol into a two-step differentiation protocol.

### ADSC preparation (Step 1)

ADSCs (StemPro® Human Adipose-Derived Stem Cells, R7788-115) from Invitrogen (Waltham, MA, USA) were cultured in accordance with the manufacturer’s guidelines. After thawing using established procedures, cells were cultured until passage 5–6, and then 5 × 10^5^ cells were seeded into 96-well ultra-low attachment plates as 3D culture or 12-well ultra-low attachment plates as conventional culture (Sigma-Aldrich Japan Co., LLC., Tokyo, Japan). For Step 1 (Fig. 1A), cells were cultured for 7 days in a differentiation cocktail of DMEM/F12 (Thermo Fisher Scientific Inc., Waltham, MA, USA), 1% human albumin (Wako, Osaka, Japan), 1% B27 supplement (Thermo Fisher Scientific), 1% N2 supplement (Thermo Fisher Scientific), 50 ng/ml activin A (Sigma-Aldrich), 10 nM exendin-4 (Sigma-Aldrich) and with (as 3D culture) or without (as conventional 2D culture) 0.1 mg/mL RCP μ-piece (FUJI FILM, Tokyo, Japan).

### Differentiation to IPCs (Step 2)

For differentiation of ADSCs in 3D or 2D culture, cells were cultured (Fig. 1A) with 50 ng/ml human hepatocyte growth factor (Funakoshi Co., Ltd, Tokyo, Japan), 10 mM nicotinamide (Sigma-Aldrich), and HDACi (valproic acid, Sigma-Aldrich) with (as 3D culture) or without (as conventional 2D culture) 0.1 mg/mL RCP μ-piece (FUJI FILM).

### Cell counts and purity

Cell number and purity were determined as described previously^4,17^. Briefly, at least three samples of the generated cells were assessed for cell number and purity by staining with dithizone dye (Sigma-Aldrich) dissolved in dimethyl sulfoxide (Burdick and Jackson, Morristown, NJ, USA) by light microscopy.

### Cell viability

Cell viability was assessed as described previously^4,17^. At least three samples of generated cells were stained using the fluorescein diacetate/propidium iodide (FDP/PI) staining kit (TAKARA Bio Inc., Tokyo, Japan) and assessed by fluorescence microscopy.

### Morphological analysis

We assessed morphological changes of IPCs using a cell fixing kit (Funakoshi). Briefly, cells were put in gels and fixed with 10% paraformaldehyde overnight, and 4 μm thick paraffin embedded sections were prepared with standard procedure. The sections were stained with haematoxylin-eosin and investigated with a microscope.

### Immunohistochemical staining

Sections were prepared after deparaffinization and antigen retrieval. Cells were pelleted by centrifugation, put on a coverslip and incubated with primary antibodies against insulin (aa287-299, LS-B129; LSBio) at a dilution of 1:100 in phosphate-buffered saline (PBS), vascular endothelial growth factor (VEGF, bs-0279R, Funakoshi) 1:200 in PBS, C-peptide (bs-0274R, Funakoshi) 1:100 in PBS for 1 h at room temperature. Cells were then incubated with biotinylated secondary antibody, followed by treatment with a streptavidin-biotin-horseradish peroxidase complex. Positive staining was visualized with diaminobenzidine and cell nuclei were counterstained with Mayer’s haematoxylin.

### Quantitative reverse transcription-polymerase chain reaction (qRT-PCR)

Total RNA was extracted from cells using a RNeasy Mini Kit (Qiagen, Hilden, Germany) and cDNA was synthesized from 2.5 µg of total RNA by reverse transcription using a SuperScript RT kit (Promega, Madison, WI, USA), following the manufacturer’s instructions and as previously described^41^. qRT-PCR was performed using the Applied Biosystems 7500 real-time PCR system, TaqMan Gene Expression Assays on demand, and a TaqMan Universal Master Mix (gene-specific TaqMan probes on a StepOne Plus; Applied Biosystems, Foster City, CA, USA). MAFA (Hs04419852_s1) TaqMan primer was used. Glyceraldehyde-3-phosphate dehydrogenase (GAPDH) was used as an internal control for normalization. Expression levels of all genes were calculated as a ratio to GAPDH. Amplification data were analysed with an Applied Biosystems Prism 7500 Sequence Detection System version 1.3.1 (Applied Biosystems).

### Cell toxicity assay

Cell toxicity of generated IPCs was investigated using the Cell Counting kit-8 (Fuji film-Wako, Osaka, Japan). Briefly, we incubated human dermal fibroblasts (Promo Cell, Heidelberg, Germany) in 24-well plates for 24 h at 37 °C after cell count. After 24 h co-culture^5^ at 37 °C with three IPCs per well (a transwell system, 0.4 μm pore size membrane; Corning, Acton, MA), the solution of Cell Counting Kit-8 was added. As negative control, we added 100 μM 5-Fluorouracil (5-FU, Wako) to fibroblast. Then, the proliferation of fibroblasts was analysed by enzyme-linked immunosorbent assay (AKRIN-011H, Shibayagi, Shibukawa, Japan) with a microplate reader at a wavelength of 450 nm.

### Glucose-stimulated insulin secretion

The glucose stimulation index (SI) of IPCs was calculated as previously described^17^. First, IPCs were cultured in RPMI-1640 medium with 5 mM glucose for 1 h and then in medium containing 45 mM glucose for 1 h, before being cultured in medium containing 5 mM glucose for an additional 1 h. The insulin concentration of the supernatant was analysed by enzyme-linked immunosorbent assay (AKRIN-011H, Shibayagi, Shibukawa, Japan) with a microplate reader at a wavelength of 450 nm. Total DNA was extracted to determine the cell count. SI was then calculated by dividing the amount of insulin secretion from the high glucose incubation by the insulin secretion from the low glucose incubation. Three independent experiments were performed.

### Electron microscopic analysis

Samples were prepared as previously reported^42^. Briefly, samples fixed with 2% paraformaldehyde and 2% glutaraldehyde in 0.1 M phosphate buffer (PB) pH 7.4 at 4 °C overnight. Samples were then washed three times with 0.1 M PB for 30 min each and postfixed with 2% osmium tetroxide in 0.1 M PB at 4 °C for 2 h. Samples were dehydrated in graded ethanol solutions as follows: 50% and 70% for 10 min, each at 4 °C, 90% for 10 min at room temperature, and three changes of 100% for 10 min each at room temperature. Samples were then treated with propylene oxide (PO) two times for 30 min each and then placed into a 70:30 mixture of PO and resin (Quetol-812; Nisshin EM Co., Tokyo, Japan) for 1 h. The cap of the tube was left open and PO was volatilized overnight. The samples were transferred to fresh 100% resin and polymerized at 60 °C for 48 h. The polymerized resins were sectioned ultra-thin at 70 nm with a diamond knife using an ultramicrotome (Ultracut UTC; Leica, Vienna, Austria) and the sections were mounted on copper grids. Sections were stained with 2% uranyl acetate at room temperature for 15 min and then washed with distilled water, followed by secondary staining with lead stain solution (Sigma-Aldrich Co., Tokyo, Japan) at room temperature for 3 min. The grids were observed by a transmission electron microscope (JEM-1400Plus; JEOL Ltd., Tokyo, Japan) at an acceleration voltage of 100 kV. Digital images (3,296 × 2,472 pixels) were taken with a CCD camera (EM-14830RUBY2; JEOL Ltd., Tokyo, Japan).

### Transplantation (*in vivo* study)

Eight-week-old nu-nu nude mice were purchased from CIEA, JAC Inc. (Tokyo, Japan) and bred at the Tokushima University animal facility. Mice were intraperitoneally injected with 200 mg/kg streptozotocin and diabetes was induced. Blood glucose levels were measured with an Accu-chek Aviva (Rossi DC Japan Inc., Tokyo, Japan) from the tail vein. The criteria for diabetes were one reading above 400 mg/dl of blood glucose or two continuous readings above 350 mg/dl. Ninety-six IPCs were transplanted under kidney capsule, intra-mesentery and intra-femoral muscle under anaesthesia with Flosen. Non-fasting blood glucose levels were measured every two days after transplantation. Recipient mice were sacrificed at day 7 and day 120 after transplantation, and extirpated organs (IPC transplanted kidneys and mesenteries) were morphologically analysed. The experiments and procedures were approved by the Animal Care and Use Committee of the University of Tokushima and were performed in accordance with the NIH Guide for the Care and Use of Laboratory Animals.

### Statistical analysis

Descriptive statistics are presented as mean ± S.D., median with range for quantitative variables and number (percentages) for qualitative variables. Univariate analysis was performed using one-way analysis of variation (ANOVA), paired and unpaired t-tests, Scheffe’s test or the Log-rank test, as appropriate. A p-value of < 0.05 was considered statistically significant and all p-values reported were two-sided. All analyses were performed with State Mate III for Windows (ATMS Co., Tokyo, Japan).

## Data Availability

The datasets used and/or analysed in this study are available from the corresponding author upon reasonable request.
